# Effect of Intraoperative Electrical Stimulation on Recovery after Rat Sciatic Nerve Isograft Repair

**DOI:** 10.1089/neur.2020.0049

**Published:** 2020-11-12

**Authors:** Galina P. Koh, Carol Fouad, William Lanzinger, Rebecca Kuntz Willits

**Affiliations:** ^1^ Biomedical Engineering, The University of Akron, Akron, Ohio, USA.; ^3^ Mechanical Engineering, The University of Akron, Akron, Ohio, USA.; ^4^ Chemical, Biomolecular, and Corrosion Engineering, The University of Akron, Akron, Ohio, USA.; ^2^ Orthopedic Surgery, Cleveland Clinic Akron General, Akron, Ohio, USA.; ^5^ Chemical Engineering, Northeastern University, Boston, Massachusetts, USA.; ^6^ Bioengineering, Northeastern University, Boston, Massachusetts, USA.

**Keywords:** electrical stimulation, histology, motor function, peripheral nerve injury, sensory function

## Abstract

Peripheral nerve injuries, associated with significant morbidity, can benefit from electrical stimulation (ES), as demonstrated in animal studies through improved axonal growth. This study combined the clinical gold standard of isograft repair in a rat model of sciatic nerve injury to evaluate the effects of intraoperative ES on functional tests and histology. Forty rats underwent a surgically induced gap injury to the right sciatic nerve and subsequent repair with an isograft. Half of these rats were randomly selected to receive 10 min of intraoperative ES. Functional testing, including response time to a heat stimulus and motor functional tests, were conducted.

Histology of the sciatic nerves and gastrocnemius muscles were analyzed after 6 and 12 weeks of recovery. Rats that underwent ES treatment showed incremental improvements in motor function between weeks 2 and 12, with a significantly higher push-off response than the no-ES controls after 6 weeks. Although no differences were detected between groups in the sensory testing, significant improvements over time were noted in the ES group. Histology parameters, sciatic nerve measures, and gastrocnemius muscle weights demonstrated nerve recovery over time for both the ES and no-ES control groups. Although ES promoted improvements in motor function comparable to that in previous studies, the benefits of intraoperative ES were not detectable in other metrics of this rat model of peripheral nerve injury. Future work is needed to optimize sensory testing in the rodent injury model and compare electrical activity of collagen scaffolds to native tissue to detect differences.

## Introduction

Peripheral nerve injury is a prevalent clinical challenge associated with high treatment costs and a significant impact on quality of life.^1^ Current clinical options yield unpredictable functional recovery for the nearly 1 billion patients worldwide who are affected by nerve trauma in the extremities.^1^ Traumatic nerve injuries range in complexity from neuropraxia, with a loss of conduction, to neurotmesis with a severe gap defect.^2^ Although neurons can spontaneously regenerate at a rate of 1 mm/day, this process may be futile for larger nerve-gap injuries. Within 12–24 months of nerve injury, the target neuromuscular end plate loses its integrity and the adjacent muscle tissue undergoes irreversible fibrotic changes. Meanwhile, sensory end organs can last up to 3 years after a peripheral nerve injury, which allows for sensory function to be recovered even though muscle function may be lost.^2,3^ Despite technological advances, the gold standard for bridging nerve-gap defects in the clinic remains the use of an autograft that provides the optimal native environment and scaffolding for axon regeneration.^2–4^ However, repair with autografts is also held to the 1 mm/day regeneration speed in humans; therefore, improvements in techniques to achieve healing prior to neuromuscular junction death continue to be investigated.

The application of an exogenous electric field can be used to augment neurite outgrowth by upregulating neurotrophic factors and promoting axonal sprouting.^5,6^ *In vitro* studies have used a pulsed electrical field of 0.1–10 V/m on chick dorsal root ganglion (DRG) cells to show improved neurite alignment and increased neurite length after 5–6 h of exposure.^7–9^ Others used direct current (DC) stimulation to provide directional cues for regenerating axons, encouraging them to grow toward the cathode, whereas a pulsed alternating current (AC) did not have the same effect.^10^ When the duration of electrical stimulation (ES) was reduced to 10 min at 24 V/m DC on chick DRG cells, the positive effects on neurite regeneration were not diminished in both two-dimensional and three-dimensional cell culture environments.^11,12^ When the ES treatment of DRG cells was compared with treatments involving cell media supplements, the ES treatment was found to increase neurite outgrowth by 40%.^13^

A rodent sciatic nerve injury model is a suitable *in vivo* method for evaluating the functional and structural repair of nerves that results from exogenous ES applied during surgery. Previous *in vivo* work has tested intraoperative ES over various time durations, ranging from 2 weeks to 1 h.^14,15^ Although 1 h proved to be effective, this increase in surgery time posed other challenges such as increased surgical costs, the risk of infection,^16^ and potential complications from prolonged anesthesia.^17–19^ In our prior work, we reduced intraoperative ES time to 10 min and showed favorable motor functional outcomes following the use of collagen-filled conduits to bridge a surgically induced nerve gap.^20^ In the present study, we sought to augment the current standard of care, auto (iso) grafts, with intraoperative ES to evaluate for possible synergistic effects. We hypothesized that 10 min of intraoperative ES after an isograft repair of a surgically induced nerve gap would enhance axonal regeneration and speed functional recovery, as evaluated by motor and sensory functional tests. To test this hypothesis in a peripheral nerve injury model, rats were randomized to receive ES on a grafted 13-mm nerve defect and functional testing was performed post-operatively over 12 weeks of recovery, whereas histology evaluations were performed after 6 weeks and 12 weeks.

## Methods

All institutional and national guidelines for the care and use of laboratory animals were followed. A total of 60 male Lewis rats were obtained from Envigo to create two groups of 20 rats per group, with an additional 20 as nerve-graft donors. Because one rat failed to recover after surgery in the control group, final group samples included *n* = 19 for the control (no-ES) group and *n* = 20 for the experimental (10 min ES) group.

From the donor rats, a segment of the sciatic nerve was excised from both hind legs, yielding 40 total nerve grafts to be used in both the experimental and control groups. The experimental group underwent a surgically induced gap injury and isograft repair of the sciatic nerve in one leg followed by 10 min of ES before wound closure. Sterile platinum electrodes were placed at the proximal and distal coaptation sites for application of 24 V/m-DC ES. Sham surgery was performed on the contralateral leg, where the nerve was exposed, but not injured. The control group underwent the same procedure on the injured and sham legs, but without the ES treatment. All surgical sites and sham incisions were closed with Michel clips. Animals were individually housed for 7 days after surgery until removal of clips, after which they were socially housed.

Rats in both groups underwent biweekly functional testing for up to 12 weeks, and 12 rats from each group were euthanized after 6 weeks for histology evaluation. The remaining 8 rats from each group continued through the full 12 weeks of testing. Functional tests included an extensor postural thrust (EPT) test, walking track analysis, and thermal sensory testing.

To perform the EPT test, a rat was wrapped in a surgical towel with one hind leg extended. The forefoot was placed in contact with the surface of a digital balance to elicit a push-off response. The largest value from the scale was recorded over a 20-sec time period. The test was repeated five times and the three largest values were used to calculate the percent motor deficit (MD%) values using the difference between the sham and experimental normalized to the sham.^21^ Largest values were chosen to measure maximal force generated from each rat.

To supplement the EPT motor function test, a walking track analysis was performed. Rats were trained pre-operatively to walk through an enclosed plexiglass walkway toward a dark shelter. Their paws were recorded on a mirror beneath the walkway. Three mid-gait screen captures were obtained for each paw, for a total of six images per rat at each time-point. Screen captures were evaluated to measure print length (PL), toe spread (TS), and the intermediate toe spread (IT) on the experimental (E) and sham (S) paws, which were then used to calculate the sciatic functional index (SFI ) value (see equation). An SFI value of −8.8 indicates complete recovery and an SFI value of −100 represents complete impairment.

$$\begin{array}{cc} SFI & =-38.3\left(\frac{EPL-SPL}{SPL}\right)+109.5\left(\frac{ETS-STS}{STS}\right)\\ & +13.3\left(\frac{EIT-SIT}{SIT}\right)-8.8 \end{array}$$

Sensory recovery was measured as a response to heat stimulus through a modified Hargreaves method.^22–24^ To conduct this test, rats were placed in a plexiglass enclosure on a glass platform, where a thermal lamp was used to focus a light beam onto the center of the plantar paw. Withdrawal time was measured with an electronic timer, which was synchronized with the thermal light. Thermal light intensity was tuned to elicit a latency response of 5–10 sec before surgery,with a cutoff exposure time of 20 sec to prevent injury. Glass temperature testing was conducted on a weekly basis to ensure consistently safe temperatures.^23^ Sensory testing was performed biweekly, four times on each rat at every time-point, with a wait period of 5 min in between trials to allow the glass to return to ambient temperature. From the four collected values, the three lowest values, indicating the quickest responses, were analyzed.

After 6 weeks of functional testing, histology samples were evaluated from 12 rats from each group, using the nerve graft from the experimental leg and the gastrocnemius muscle from both the experimental and sham legs. After 12 weeks of functional testing, histology samples were evaluated from the remaining 8 rats from each group. Muscle weights were recorded during the tissue harvest and used to calculate the relative muscle mass ratio of the injured to sham leg, dividing the weight of the experimental muscle by the weight of the sham muscle.

All tissue samples were fixed in 4% paraformaldehyde for 48 h and stored in phosphate buffered saline containing 0.02% sodium azide at 4°C until processing. Muscle and nerve samples were then post-fixed in 4% glutaraldehyde, embedded into paraffin blocks, and cut into 4-μm slices. Muscles were sectioned at the estimated midline and stained with hematoxylin and eosin (H&E). Nerve grafts were stained with osmium tetroxide and cross-sectionally sliced in three locations: 2 mm proximal to the coaptation site, at the estimated midline, and 2 mm distal to the coaptation site, resulting in three sections from each nerve graft. A slide scanner was used to image all tissue sections at 20 × magnification. To quantify images, three regions of interest (ROIs), measuring 300 × 300 pixels in ImageJ, were randomly selected by blinded investigators for analysis. Nerve ROI analysis included the total number of axons, axon area, and axon diameter within a known image area (2.22 × 10^−2^ mm^2^). Muscle images were qualitatively assessed.

### Statistical analysis

To evaluate differences within each group over time for the functional evaluations, a repeated measures analysis of variance (ANOVA) was performed on the 12-week group for functional test data, where *p* < 0.05 was considered significant. To compare the two experimental groups, a one-way ANOVA was performed at each of the time-points on the same data sets. Because such an analysis is subject to error through multiple comparisons, a conservative Bonferroni correction was applied, which lowered the statistical significance threshold to *p* < 0.008 for functional tests between groups at each time-point. Muscle ratio was evaluated using a two-way ANOVA (independent variables of time-point and experimental group) with a Tukey post hoc test, where *p* < 0.05 was considered significant. Nerve histology metrics were evaluated using a one-way ANOVA to evaluate differences between groups in one analysis and to evaluate differences between time-points in a second analysis, where *p* < 0.05 was considered significant.

## Results

### Extensor postural thrust

**FIG. 1.**
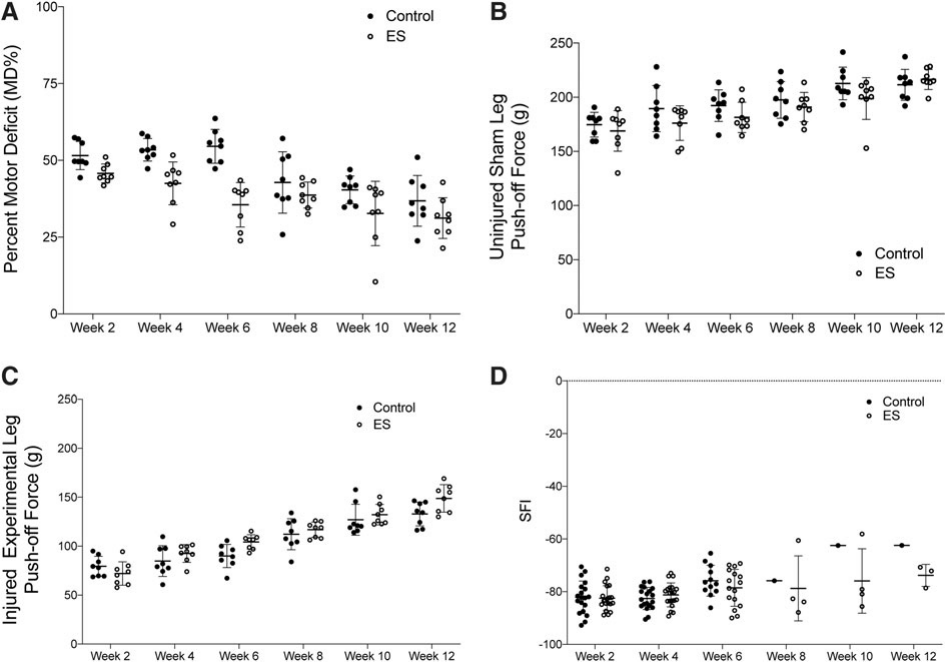
Motor function was evaluated by EPT **(A–C)** and SFI **(D)**. (A) MD%, a normalized value of injured to sham leg response of each rat, showed a decreasing trend over the 12 weeks as motor function improved. Data from 12-week animals are reported on the figure. Statistical analysis on the 12-week animals showed an MD% decrease between weeks 4 and 10 (control, *p* = 0.002; ES, *p* = 0.05), 4 and 12 (control, *p* = 0.003; ES, *p* = 0.002), and between weeks 6 and 10 (control, *p* = 0.013; ES, *p* = 0.008). Additionally, the control group showed MD% differences between weeks 4 and 8 (*p* = 0.009), weeks 6 and 8 (*p* = 0.035), and weeks 6 and 12 (*p* = 0.044). The ES group showed MD% differences between weeks 2 and 12 (*p* < 0.001). (B) Push-off force of the uninjured sham leg showed a consistency in data collection over the 12-week testing period with no differences between the control and ES groups at any of the time-points. However, some differences were found between time-points within the groups, week 2 and 6 in the control group (*p =* 0.039), between weeks 2 and 12 (control, *p* = 0.005; ES, *p =* 0.001), and between weeks 6 and 12 in the ES group (*p =* 0.021). (C) As expected, push-off force of the injured experimental leg showed a steady increase over the 12-week testing period, with differences between week 2 and 6 in the ES group (*p* < 0.001), between weeks 2 and 12 (control, *p =* 0.001; ES, *p* < 0.001), and between weeks 6 and 12 (control, *p =* 0.001; ES, *p =* 0.002). The ES group showed a significantly higher push-off force than the control group at the 6-week time-point (*p =* 0.005). Although the push-off averages were consistently higher in the ES group than in the control group (*p =* 0.03 at week 12), no further differences were found between the groups at any of the time-points with a conservative Bonferroni correction to adjust for multiple comparisons. (D) SFI was determined every 2 weeks for each group. All animals except one in the control group and three in the ES group developed contractures, and therefore, the paw print could not be accurately tracked. Contractures is a common problem with SFI, which is why both EPT and SFI were used to track motor function. EPT, extensor postural thrust; ES, electrical stimulation; MD%, percent motor deficit; SFI, sciatic functional index.

Motor function was evaluated biweekly after surgery in 12 rats per group over 6 weeks of testing and in 8 rats per group over 12 weeks of testing. To investigate the repair over the course of the 12 weeks, data reported are focused on the 12-week groups. A comparable recovery of motor function was observed in both the control and ES groups through an overall decreasing trend in MD% over 12 weeks, with consistent standard deviations in each group over the 12 weeks of testing (Fig. 1A). Although no differences were found between the two groups at any of the time-points, both groups showed incremental recovery through significant decreases in motor impairment over weeks 4 and 10 (control, *p =* 0.002; ES, *p* = 0.05), 4 and 12 (control, *p* = 0.0003; ES, *p =* 0.002), and 6 and 10 (control, *p* = 0.013; ES, *p* = 0.008). The ES group showed an additional decrease in motor impairment over weeks 2 and 12 (*p* < 0.001; Fig. 1A). The push-off response of the injured leg showed consistently higher values in the ES group (*p* = 0.005 at 6 weeks, *p* = 0.03 at 12 weeks) than in the no-ES control (Fig. 1C), whereas the non-injured leg showed a similar response between the two groups over time (Fig. 1B).

### Walking track analysis

Another measure of motor function is the walking track analysis, which is comparable to the EPT test, except that it tends to have higher standard deviations and a risk of losing data to contractures forming in the paws.^21^ Contractures are a well-known complication in peripheral nerve injury models stemming from an imbalance of innervation between the flexor and extensor muscle groups.^25,26^ Some rats did not have a measurable paw print after week 6 because of severe contractures in their paws and were excluded from the analysis. In the control group, 52% of the rats developed contractures compared with 45% in the ES group over the 12-week testing period. After 6 weeks, the control group had a sample size *n* = 1 because of contracture complications (Fig. 1D). Although the general recovery trend was similar in both groups over 12 weeks, with SFI values trending upward from −100 toward −60 over time, a statistical analysis could not be completed with this incomplete data set.

### Sensory functional testing

**FIG. 2.**
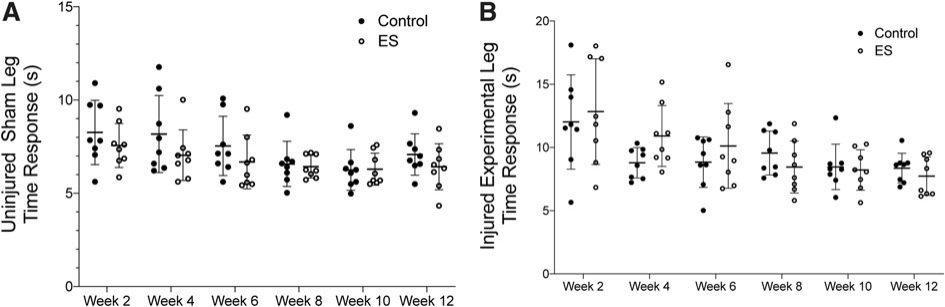
Sensory function. **(A)** For animals tested over 12 weeks, sensory response of the uninjured sham leg, the paw-lift response to a thermal stimulus, was consistent over the weeks of data collection and in the range of 5–10 sec, which was the pre-surgery latency response time. No statistical differences were found between groups or time-points. **(B)** Sensory response of the injured experimental leg showed a loss of function at week 2, with higher sensory response times, and a steady regaining of function with sensory response times decreasing throughout the 12 weeks of recovery. Differences were found in the ES group between weeks 2 and 6 (*p =* 0.037), weeks 2 and 8 (*p =* 0.03), weeks 2 and 10 (*p =* 0.016), weeks 2 and 12 (*p =* 0.005), weeks 4 and 8 (*p =* 0.002), weeks 4 and 10 (*p* < 0.001), and weeks 4 and 12 (*p =* 0.002). ES, electrical stimulation.

Sensory recovery was evaluated by a paw-lift response to a heat stimulus through the duration of the study; 6 weeks for 12 rats per group and 12 weeks for the remaining 8 rats per group. A latency value was established pre-surgery to serve as the 5- to 10-sec response baseline, which was maintained in the sham leg of rats in both groups, control and ES, throughout the 12 weeks of testing (Fig. 2A). A decreasing trend in paw withdrawal time was noted in both the control and ES groups over the 12-week testing period, with differences noted in the ES group between weeks 2 and 6 (*p* = 0.037), weeks 2 and 8 (*p* = 0.03), weeks 2 and 10 (*p* = 0.016), weeks 2 and 12 (*p* = 0.005), weeks 4 and 8 (*p* = 0.002), weeks 4 and 10 (*p* < 0.001), and weeks 4 and 12 (p = 0.002; Fig. 2B). Although no differences were found between the control and ES groups, incremental differences between weeks in the ES group showed steady signs of recovery.

### Histology of muscle and nerve

**FIG. 3.**
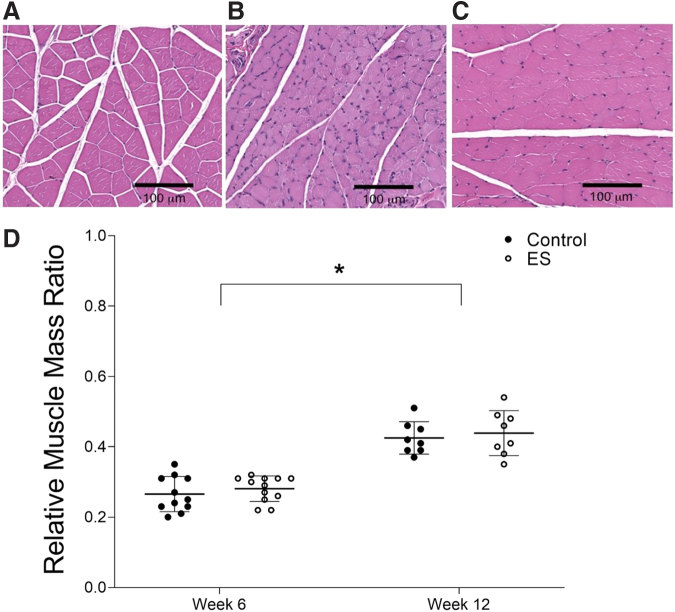
Muscle histology. **(A)** Healthy muscle fibers, as shown by a representative section with an H&E stain from the sham leg of a rat in the 6-week ES group. Muscle fibers are clearly defined, with purple nuclei. All muscle image scale bars are 100 μm. **(B)** Degenerated muscle fibers, as shown by a representative section from the injured leg of a rat in the 6-week ES group. Muscle fibers show signs of shrinking from muscle degeneration, with more dense nuclei per sampling area. **(C)** Visible regeneration, as shown by a representative section from the injured leg of a rat in the 12-week ES group. Muscle fiber size increased from week 6, with a less dense nuclei per sampling area. **(D)** Relative muscle mass ratio, a normalized value of the injured leg muscle weight to the sham for each rat, showed a significant increase between weeks 6 and 12 for both the control and ES group (*p* < 0.001). * denotes *p* < 0.05. ES, electrical stimulation; H&E, hematoxylin and eosin.

The gastrocnemius muscle is innervated by the sciatic nerve, allowing for a comparison of nerve reinnervation between groups. Using the sham and surgical leg muscle weights to calculate the relative muscle mass ratio, we found a significant increase between weeks 6 and 12 (*p* < 0.001) in both the control and ES groups, indicating a similar reinnervation trend (Fig. 3D). Histology images support these findings by showing a similar muscle degeneration pattern between both groups in muscle sections from the injured leg, with shrunken muscle fibers and more dense nuclei per sampling area (Fig. 3B). After 12 weeks, signs of regeneration were visible in the injured muscle sections from both groups, with larger muscle fibers and more disperse nuclei (Fig. 3C). The sham leg muscle sections from both groups had a consistently healthy appearance of muscle fibers and nuclei between weeks 6 and 12 (Fig. 3A).

**FIG. 4.**
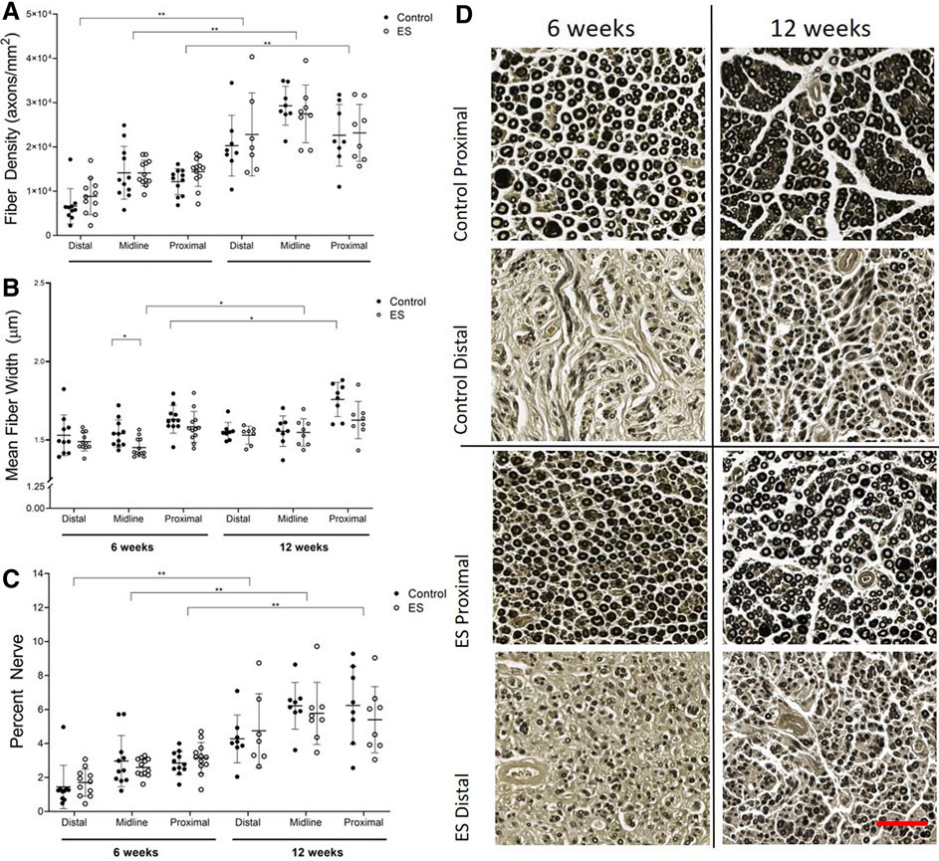
Nerve histology. **(A)** Fiber density, a measure of the total number of axons per sampling area, was calculated for all nerve sections, taken from the distal, midline, and proximal segments of the injured-leg nerve of each rat. Although there were no differences between the groups, both groups showed a significant increase in fiber density between 6 and 12 weeks in the distal, midline, and proximal segments (*p* ≤ 0.001). **(B)** Mean fiber width, a measure of the axons showing maturity of the regenerating fibers, was calculated for all nerve sections taken from the distal, midline, and proximal segments of the injured-leg nerve of each rat. Differences were found between weeks 6 and 12 in the ES group at the midline segment (*p =* 0.009) and in the control group at the proximal segment (*p =* 0.012). A difference was found between the ES and control groups in the midline sections of the 6-week time-point (*p =* 0.009). **(C)** Percent nerve, a measure of the axon area relative to the sampling area, was calculated for all nerve sections taken from the distal, midline, and proximal segments of the injured-leg nerve of each rat. Differences were found between weeks 6 and 12 in the midline and distal segments (*p* ≤ 0.001) and in the proximal segment (control, *p* < 0.001; ES, *p =* 0.003). **(D)** Representative nerve sections at both time-points, 6 weeks and 12 weeks, with samples from ES and control groups at the distal and proximal segments of the nerve, stained with osmium tetroxide. Early degeneration of the nerve can be observed at the 6-week time-point in the difference between the distal and proximal sections, where the proximal end has a healthier appearance with clearly defined black myelinated axons. Regeneration can be observed in comparing the 12-week distal segments with their 6-week counterparts, where the number of visible axons increases. This is supported by quantified data, where there are significant increases of fiber density, percent nerve, and fiber width between weeks 6 and 12. Scale bar for all images is 50 μm. ES, electrical stimulation.

Nerve histology was performed on graft explants from the injured leg of rats from both control and ES groups, with sections taken from the distal, midline, and proximal regions of the graft. The number of myelinated axons per sampling area were quantified and used to calculate the fiber density to compare differences between groups and harvest time-points. Although no differences were found between the groups, there was a significant increase in fiber density between weeks 6 and 12 in both the control and ES groups across all three segments of the graft (*p* < 0.001; Fig. 4A). Additional measures of nerve maturity are mean fiber width and percent nerve. Mean fiber width of quantified axons showed differences between weeks 6 and 12 in the ES group at the midline segment (*p* = 0.009) and in the control group at the proximal segment (*p* = 0.012). In addition, a difference in mean fiber width was found between ES and control groups in the midline sections of the 6-week time-point (*p* = 0.009; Fig. 4B).

Percent nerve measures showed differences between weeks 6 and 12 for both control and ES groups at the midline and distal segments (*p* ≤ 0.001) and at the proximal segment (control, *p* < 0.001; ES, *p* = 0.003; Fig. 4C). Initial nerve degeneration after injury was visible in the 6-week time-point when comparing the distal and proximal segments. The distal segments showed visibly less osmium-stained myelinated axons than the proximal segments. After 12 weeks, regeneration was visible in a comparison of the distal segment between weeks 6 and 12, where there was a visible increase in the number of myelinated axons in the distal segments of both the control and ES groups after 12 weeks of recovery (Fig. 4D). Average metrics of quantified nerve sections from both groups at both time-points are noted in Table 1.

**Table 1.** Average Metrics of Quantified Nerve Sections from ES and Control Groups at 6 Weeks and at 12 Weeks

Nerve histology metric	6 weeks						12 weeks					
	Proximal		Midline		Distal		Proximal		Midline		Distal	
	ES	Control	ES	Control	ES	Control	ES	Control	ES	Control	ES	Control
Fiber density (axons/mm^2^)	14,391.41	12,149.37	14,118.22	14,137.81	8016.78	7076.24	23,167.99	22,620.98	27,467.93	29,285.66	23,317.74	20,275.99
% Nerve	3.15	2.85	2.60	2.96	1.56	1.55	5.40	6.24	5.77	6.22	4.85	4.27
Mean fiber width (μm)	1.58	1.63	1.45	1.54	1.49	1.53	1.63	1.76	1.55	1.56	1.53	1.55

ES, electrical stimulation.

## Discussion

Electrical activity naturally occurring in the peripheral nerves has sparked many research questions to investigate how exogenous electrical therapy can be used to encourage nerve growth after injury. From such investigations, the literature shows that intraoperative ES influences nerve regeneration through increased neurite length, directional alignment of growing neurites, and the upregulation of biochemical factors to support growth.^5,6,9,27,28^ In contrast, some earlier work using ES *in vivo* for nerve regeneration demonstrated deleterious effects,^29,30^ likely related to electrochemical byproduct production at the electrodes. In the intervening years, it has been shown by multiple groups that ES can be applied in a supportive manner for nerve regeneration and that considerations for applied field voltage, current, duration, and electrode material choice and positioning are necessary to realize the effects.^31,32^ ES can be applied using various methods, including both DC electrical fields, as applied here, or alternating or pulsed current fields, as has been applied with both previous *in vivo* one-time stimulation studies and with implanted devices.^14,15,31–35^ The alternating paradigm provides a charge balance between the anode and cathode to reduce any electrochemical reactions that can occur at the electrode with one-directional current.

However, new approaches to apply *in vivo* implantable DC fields have been developed,^35^ and it has been noted that an advantage of DC application is that it can both excite and inhibit neuronal activity. Our lab previously demonstrated that the intraoperative ES time can be shortened from 60 min to 10 min, while still producing a beneficial effect on motor response in a collagen scaffold graft. Although we have previously reported the effects of DC ES on acellular conduit repair, we further needed to demonstrate the potential effect of this DC electrical field on cellular repair methods, such as an autograft. Therefore, the current study was motivated by previously published ES benefits to assess whether a short duration of ES could enhance both motor and sensory recovery when coupled with the accepted gold standard of care of a human nerve autograft, as modeled in the rat isograft.^20^

The sciatic functional index (SFI), using walking track analysis, is a functional test that has been widely used as a standard to quantify motor recovery in rodent models.^21,25,36–39^ However, studies reported in the literature have demonstrated that this measure tends to be less reliable in early weeks due to high standard variations and paw contractures.^40,41^ In the current study, contractures began to form at week 6 and were sustained through the 12 weeks of testing, leaving us with an incomplete data set in later weeks. Accounting for this factor beforehand, our study utilized EPT as a supplemental measure, as EPT is another measure of motor functional recovery in rodent models^21,26,36^ that has been found to correlate well with SFI results.^21^ Both functional assays showed an upward trend of restoring motor function over time, without significant differences between the control and ES groups.

As expected, data from the EPT test had lower standard deviations and a complete data set throughout the 12 weeks of testing despite contractures forming after week 6. Whereas others found an average 20% decrease in MD% after 2 months of recovery after isograft repair,^36^ our data showed close to a 20% decrease in MD% after 12 weeks of testing in the ES group. When considering the raw push-off response, our results are consistent with our previous work, where the force was an average of 80*g* in both groups after 2 weeks post-op and incrementally increased to an average of 140*g* after the full 12 weeks of recovery.^20^ Although a significant difference was found only at the 6-week time-point, the ES group showed consistently higher averages in raw push-off force of the injured leg when compared with the injured leg from the control group. The push-off force showed some differences between the groups, where the MD% analysis did not, which may suggest that the benefit from ES may be too small for detection with some motor functional evaluations.

Sensory testing is of interest in hand surgery due to its relevance in protective sensation, which motivated its inclusion in this study. Detecting a sensory response in rodents poses technical difficulties, as the sensory stimulus needs to be accurately targeted and a response needs to be accurately differentiated from normal rodent behavior. The literature describes some functional assays for whole-paw stimulus, such as in a hot water bath^36^ or a nociceptive response to electrical stimuli,^42^ which are less effective in evaluating the more specific responses characteristic of sciatic nerve recovery.^43^ The Von Frey filament test and Hargreaves thermal sensory stimulus test rely on a targeted specific placement of stimulus on the paw and are more suitable for evaluating sciatic nerve injury.^22^ We chose to use the Hargreaves thermal method, which showed a consistent response in the sham leg, clear signs of injury with an impaired response of the injured leg, and a steady decrease in response time to show recovery in the injured leg over time. Whereas the sham leg from both groups had a consistent response time between 5 and 10 sec, the established latency period, the injured group had an average response time between 10 and 15 sec after 2 weeks, which decreased below 10 sec after 12 weeks.

Although contractures began to form after 6 weeks of testing, the response time did not show any drastic changes between weeks 6 and 8, indicating that the contractures did not negatively influence our ability to collect the sensory data. Sensory testing for peripheral nerve injuries in a rodent model is a challenge because it is less standardized in the literature and data collection can be more subjective, although we controlled for this by having the same person collect all of the sensory data. More work needs to be done to establish which testing method is more suitable for detecting sensory recovery after peripheral nerve repair in rodent models so that it can be included in future nerve recovery work, with the aim of restoring protective sensation in humans after a peripheral nerve surgery.

Measures of functional recovery, both sensory and motor, were only sensitive to ES when evaluating recovery over time but not when compared with the control at each of the time-points. Previously, implanted continuous ES was used on a sciatic nerve crush injury, where small enhancements in recovery occurred 2–4 days earlier in the ES group than in the control group.^44^ It would be difficult to assess such small changes in functional recovery in our model, if they had occurred, due to weekly testing time-points. However, the similarity in functional responses between the groups agreed with the literature in showing that ES did not negatively affect the motor and sensory recovery of the nerve.^45^

To investigate the effect of ES at the tissue level, nerve regeneration and muscle atrophy were assessed through histology evaluations. Percent nerve and mean fiber width are parameters that indicate the quality and maturity of regenerating nerve fibers.^46^ Both of these evaluations were similar between the control and ES groups, suggesting that short-term ES did not adversely affect the regenerating nerves, which is consistent with a study published in the literature that demonstrated no adverse effects within the nerve histology 3 days post-operative after a 1-h procedure using intraoperative ES.^45^ Although muscle atrophy was observed at the 6-week time-point and significantly reduced after 12 weeks, ES did not show any significant effect on muscle atrophy compared with the control.

After nerve injury, the distal end of the nerve undergoes Wallerian degeneration, which explains why the midline is often used in studies to show axonal growth.^47^ Our study utilized the proximal segment, in addition to the distal and midline, for comparison along the full length of the nerve. This evaluation allowed us to detect that the average fiber density of the proximal segment after 6 weeks was consistent with that in the literature for the fiber density of the average healthy rat peripheral nerve,^48^ suggesting that the proximal end of the nerve had less degeneration than other segments. In the distal and midline sections from the current model, using a 13-mm nerve-gap injury fiber density, were found to be consistent and comparable with that in the literature for isograft repair after a 10-mm nerve-gap injury,^20,49^ suggesting that the ES did not inhibit nerve repair.

Although prior literature shows increased axonal outgrowth in the presence of ES,^15,20,33^ our results showed that the increases in fiber density of the ES group was comparable with the control between the two time-points. Previous work demonstrated that exogenous ES could increase axonal growth *in vitro*^5–9^ and promote preferential reinnervation of motor and sensory pathways *in vivo.*^14^ From prior work in our lab, we saw that nerves can grow through 10-mm collagen scaffolds within 12 weeks and show significant motor recovery after only 10 min of intraoperative ES.^20^ The current study aimed to synergistically combine these previously found benefits from 10 min of ES together with a clinical gold standard by using the isograft nerve injury model in a larger 13-mm gap injury. However, we found that the same motor recovery did not translate from our previous work to the current study, and that the sensory data were not sensitive enough to elucidate the differences between our control and experimental groups.

The differences noted here, compared with previous reports, could be due to the differences in the graft itself. Previous reports have focused on cell-free scaffolds, whereas this report focused on a living cell-based scaffold. It would be interesting to further directly compare cell-free and cell-based scaffolds to examine if the cellular infiltration step of the regenerative process is supported by ES. Either way, we demonstrated that the application of ES is feasible, but as tested, does not add significant advantages within the isograft repair model. Future work is needed to compare the electrical activity of rat sciatic nerve tissue and collagen scaffolds to further probe whether this may have contributed to the differences in nerve growth, specifically the axon count within the nerve grafts and the motor functional recovery differences after 12 weeks of post-operative recovery.

## Abbreviations Used

ANOVA: analysis of variance
DC: direct current
DRG: dorsal root ganglion
EPT: extensor postural thrust
ES: electrical stimulation
H&E: hematoxylin and eosin
IT: intermediate toe spread
MD%: percent motor deficit
PL: print length
ROI: region of interest
SFI: sciatic functional index
TS: toe spread
